# Therapeutic use of psychedelic drugs in depression disorders

**DOI:** 10.1192/j.eurpsy.2021.2062

**Published:** 2021-08-13

**Authors:** M. Trigo

**Affiliations:** Psychiatry, Centro Hospitalar Universitário do Algarve, Faro, Portugal

**Keywords:** depression disorders, psychedelic drugs, LSD, Psychopharmacology

## Abstract

**Introduction:**

Depression is one of the most prevalent mental illnesses, leading to important personal distress and economic consequences. Treatment is long, often involving psychotherapy and pharmacological treatment, and relapses are frequent. Used mostly for treatment of mood disorders and alcohol dependence, drugs such as lysergic acid diethylamide (LSD) were studied in the 1950’s and showed therapeutic promise in attenuating depressive symptoms. However, in the 1960s all major psychedelic research programs were ended. Recently, there is a renewed research interest in these drugs, considering its antidepressant potential.

**Objectives:**

To review current knowledge on the therapeutic uses of psychedelic drugs such as LSD in depression disorders.

**Methods:**

Review of the most recent literature regarding the therapeutical potential of psychedelic drugs such as LSD in depression disorders. The research was carried out through the UptoDate, PubMed, MedLine, ScienceDirect and SpringerLink databases, using the terms “LSD”, “psychedelic drugs” and “depression disorders”, until December 2020.

**Results:**

As in past scientific studies, data of recent clinical research shows that the use of LSD relieves distress concerning death, particularly in terminally ill oncologic patients, and addictions including alcoholism and nicotine. There is more limited data concerning the use of classic hallucinogens to treat depression and anxiety disorders.

**Conclusions:**

Although research has shown many of the neurobiological and psychological effects of classic hallucinogens on humans, the studies that have been completed to date are not sufficient to establish clinically relevant effects. Despite further research is needed, the outcomes are encouraging, and larger, well-designed, placebo-controlled trials are now underway or being planned.

**Disclosure:**

No significant relationships.

